# Supplementary material for: Myosin 7 and its adaptors link cadherins to actin
Source: Nat Commun. 2017 Jun 29;8:15864. doi: 10.1038/ncomms15864 (PMC5493754; doi:10.1038/ncomms15864)
Supplement: Supplementary Information — Supplementary figures, supplementary tables and supplementary references. [file ncomms15864-s1.pdf]

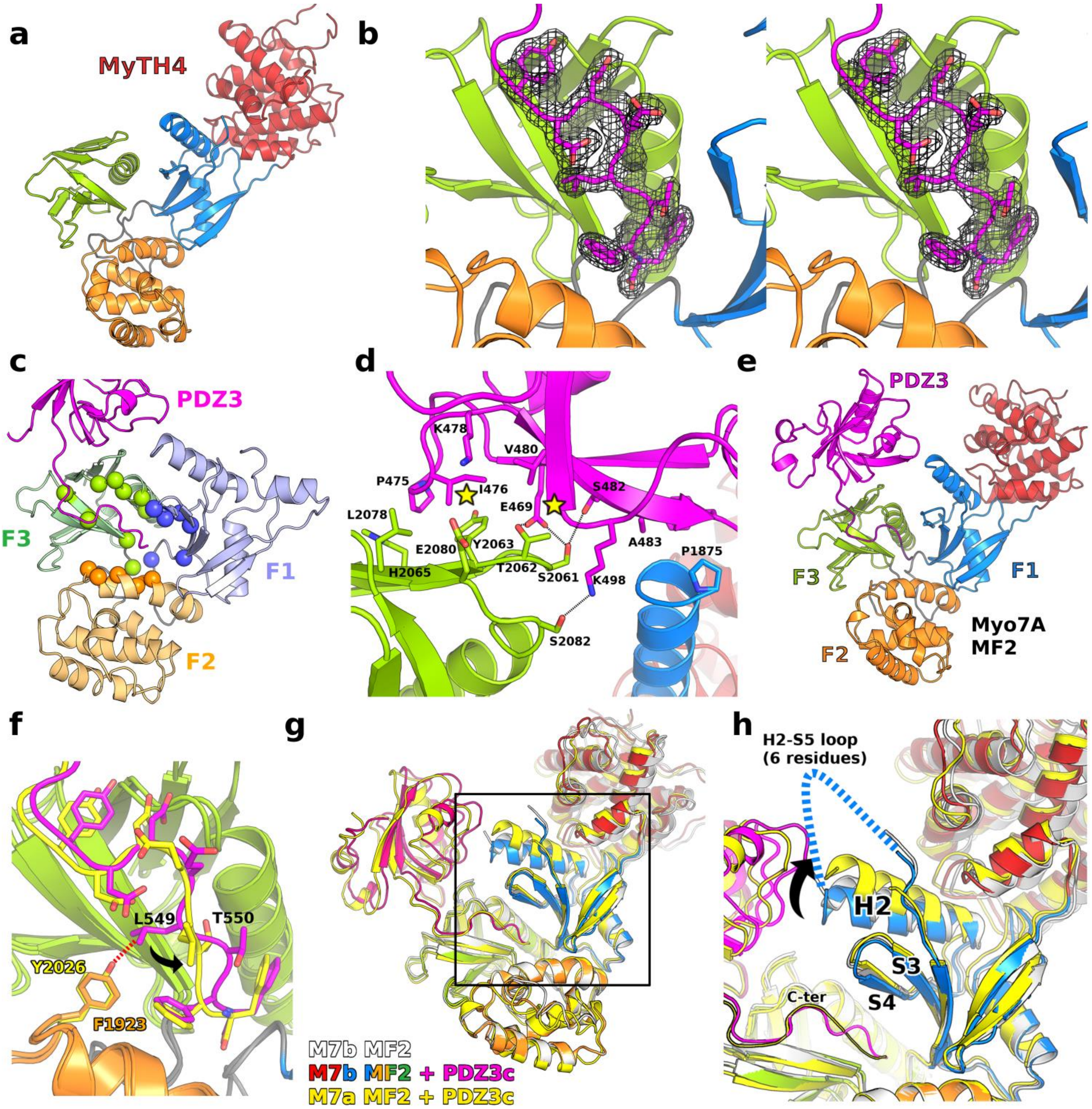

Supplementary Figure 1 – Details of the Harmonin PDZ3 recognition by Myo7 MF2.

(a) Ribbon representation of the Myo7b MF2 structure. **(b)** Stereo image of the binding pocket for the PDZ3c/Myo7b MF2 structure. The $2F_O$-F_C density map of PDZ3 C-terminal extension contoured at 1σ is shown in black. **(c)** Residues that define the groove specificity for polypeptide chain binding in the FERM domain are shown with balls (colored according to the lobe they belong to). **(d)** Interface between the harmonin PDZ3 domain (magenta) and the Myo7b MF2 F1 (blue) and F3 (green) lobes. Similar interactions are also formed in the Myo7a MF2/PDZ3c structure. The stars indicate the mutated PDZ3 residues E469 and I476. **(e)** Ribbon representation of the structure of the harmonin PDZ3c/Myo7a MF2 complex. Binding of PDZ3c to Myo7a MF2 results in the buried solvent accessible surface area of 1135 $Å^2$ (400 $Å^2$ from PDZ3 domain and 735 $Å^2$ from Cter). **(f)** Structural basis of harmonin PDZ3c binding to Myo7a and Myo7b MF2. The C-ter extension (Myo7b in magenta, Myo7a in yellow) adopts a different conformation (black arrow) in order to accommodate the Y2026's extra hydroxyl group (the steric clash is indicated with a red dash line). **(g)** Superimposition of Myo7b MF2 free (white), PDZ3c/Myo7b MF2 (multicolored) and PDZ3c/Myo7a MF2 (yellow) using the F2 lobe as a reference. **(h)** Details of the structural comparison of the boxed region shown in (g) centered on the F1 lobe. Note that the relative position of the F1 (blue)/F2 (orange) lobes is conserved. In contrast, variability within the F1 lobe (H2 helix, S3 and S4 strands and the loop that connects them) can impact the position of elements that face the central FERM cavity. These elements vary between the three structures (black arrow), and can fine-tune the binding site of the FERM groove, modulating the interactions in the groove. The H2-S5 loop (missing in the three crystal structures) is represented with a dash line.

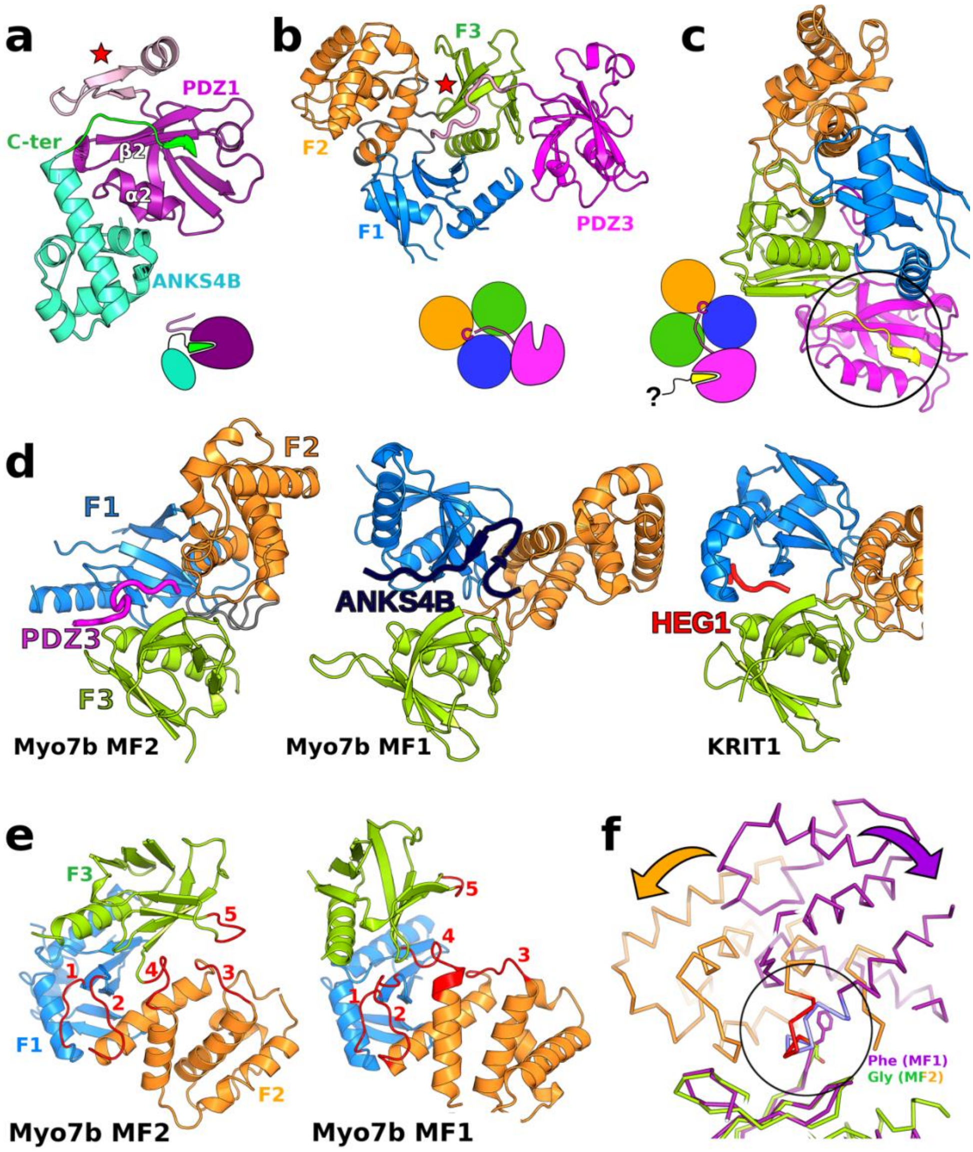

Supplementary Figure 2 – Different modes of binding for PDZ and MyTH4-FERM domains

(a-c) Examples showing the importance of the N- or C-ter extension of PDZ domain in partner recognition. **(a)** The structure of harmonin NPDZ1 and SANS SAM-PBM complex (3K1R) showing the Cter extension (light pink, red star) of harmonin PDZ1 (dark purple) folds into a minidomain and together with PDZ1 form a groove in which the SANS SAM domain and its Cter extension (containing the PBM, green) fit specifically and form strong interactions[1]. **(b)** The Cter extension of harmonin PDZ3 (magenta) uses a different binding mode to interact with Myo7b MF2, as determined in this study. In this case the Cter motif extends away from the PDZ fold and binds strongly within the FERM lobes while few interactions form between the FERM and the PDZ3 domains. **(c)** The harmonin PDZ3 canonical binding groove (circle) remains free to interact with other proteins, even after formation of the Myo7b MF2/PDZ3c complex. The PDZ3 domain is predicted to belong to Class II PDZ and the tail of another protein (yellow) may thus be accommodated[2]. **(d)** Representation of the FERM domain of Myo7b MF2 (left, this study), Myo7b MF1 (center, 5F3Y) and KRIT1 (right, 4HDQ) bound to different partners. The F3 lobe (green) is shown in the same orientation while the position/orientation of the F1 and F2 lobes differs greatly. Note that the three partners take advantage of the FERM central cavity, but PDZ3c goes deeper and interacts with all three lobes. **(e)** Location of the five linkers (red) that determine the relative position of the three lobes of the FERM domain shown in Myo7b MF2 (left) and Myo7b MF1 (right) with the F1 lobe in a similar orientation. The sequence of the linkers is variable (Supp Fig. 3) and in large part dictates the lobe orientation. **(f)** Comparison of the F2/F3 interface of Myo7b MF2 (green/orange) and MF1 (purple), with F3 lobes aligned. A significant sequence change in linker 4 (blue (MF1)/red (MF2)) is shown (Phe1387$_{MF1}$/Gly2008$_{MF2}$). As a consequence, the F2 lobe orientation changes drastically relative to the F3 lobe (arrows).

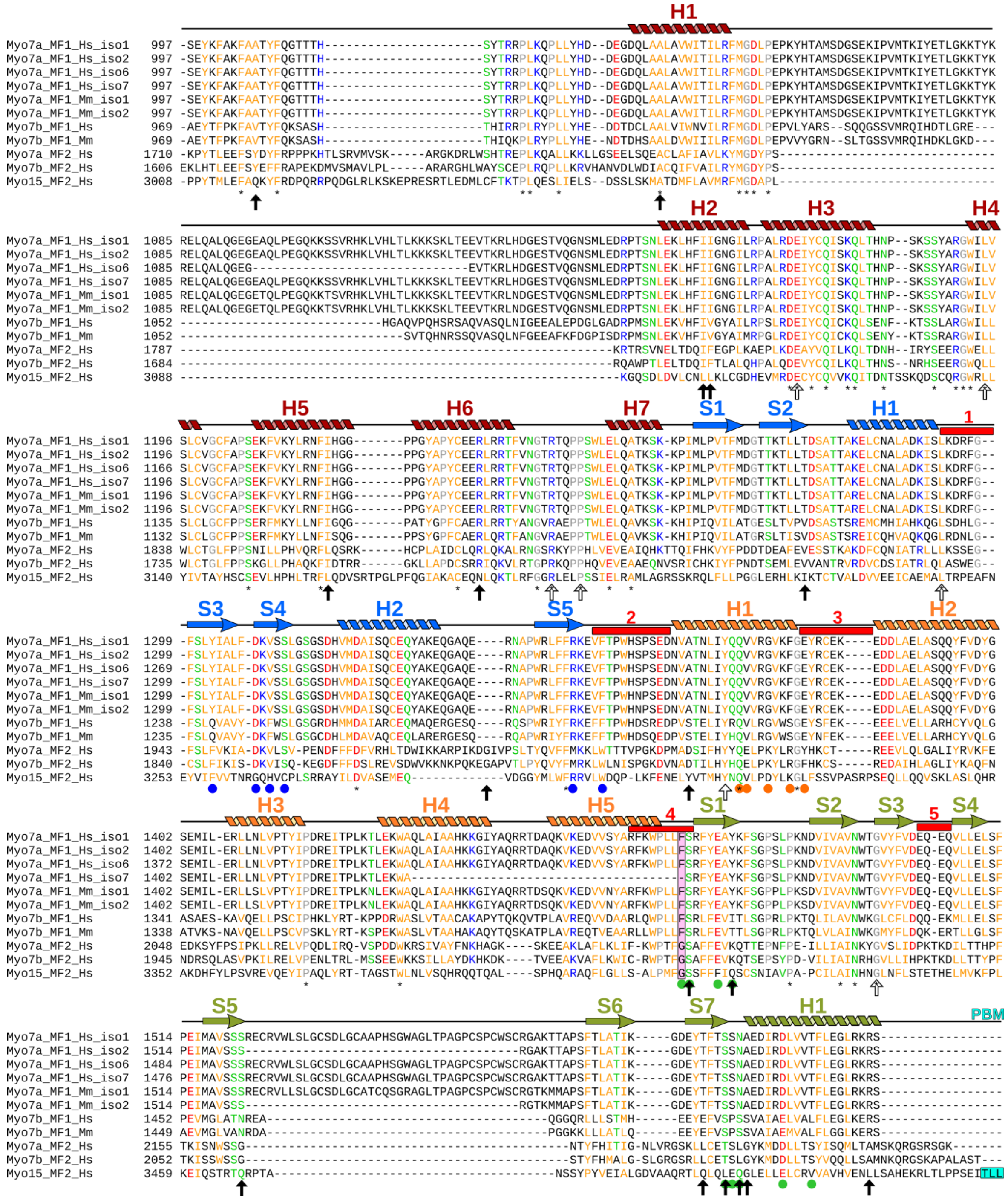

Supplementary Figure 3 – Comparison of FERM domains from Myo7 and Myo15

Structure based alignment MF1 and MF2 domains of human and mouse Myosin 7a (different isoforms), 7b and 15. Highly conserved residues are colored in yellow (hydrophobic), blue (positively charged), red (negatively charged), green (polar) and grey (glycine and proline). The linkers that dictate the relative position of the FERM lobes are indicated with red bars (1 to 5). Blue, orange and green spheres correspond to residues that define the internal pocket in the FERM domain (Supplementary Fig. 1C). Highlighted in pink in linker 4 are the phenylalanine (MF1) and the glycine (MF2) residues found between the F2-H5 and the F3-S1 elements that are crucial for the relative position of the FERM lobes (Supplementary Fig. 2F). Black and white arrows indicate the missense mutations found in the MF2 of Myo7a (Fig. 4C) and analysed in Supplementary Table 3; while the white arrows specify residues that are strictly conserved between the aligned sequences. Hs: *Homo sapiens*; Mm: *Mus musculus.* Sequences used for analysis are available under UNIPROT accession numbers Q13402 (Myo7a Hs), P97479 (Myo7a Mm), Q6PIF6 (Myo7b Hs), Q99MZ6 (Myo7b Mm) and Q9UKN7 (Myo15 Hs).

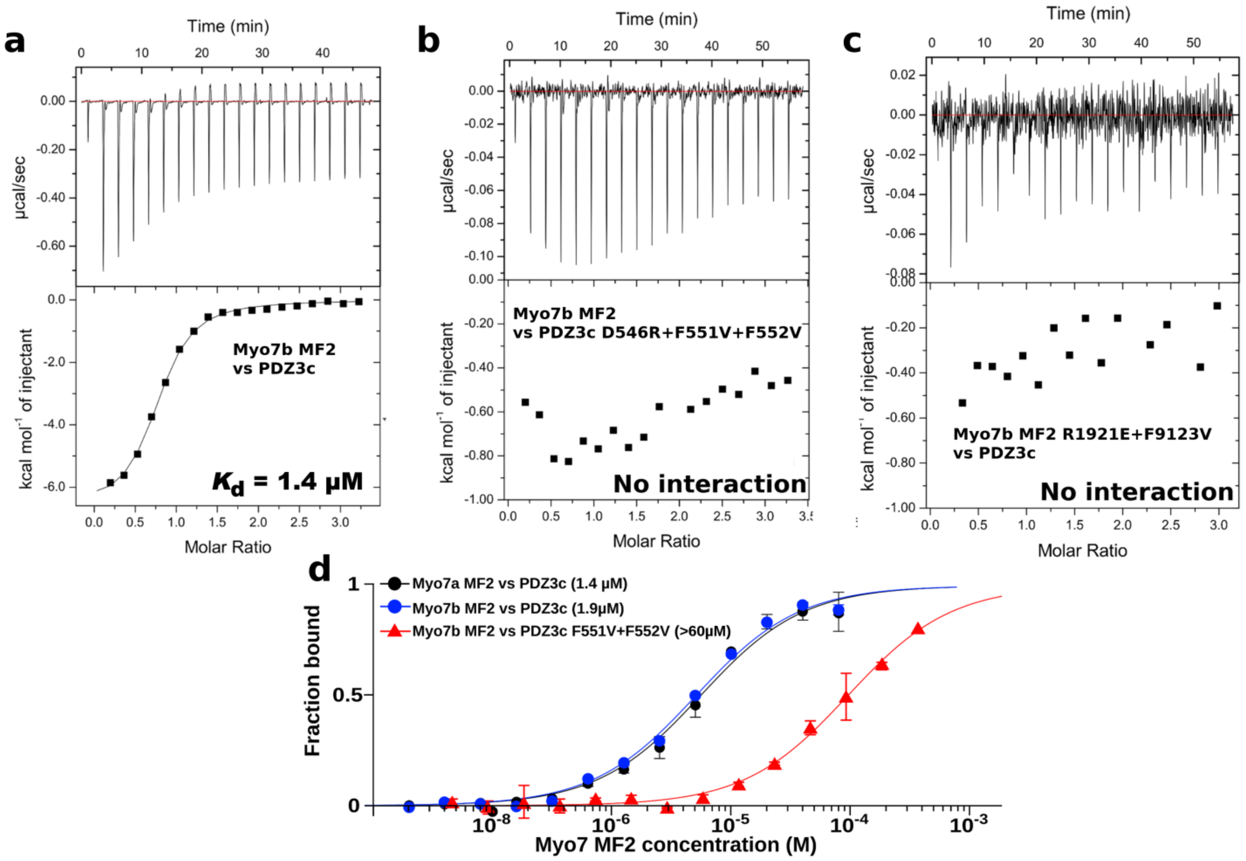

Supplementary Figure 4 – Direct recognition of Harmonin by the Myo7 MF2 domain

Representative ITC **(a-c)** and MST **(d)** binding curves showing the interaction between PDZ3c and Myo7 MF2 and the effects of mutations on the interaction. Details on the temperature and buffers used are in Materials and Methods. The average of two technical replicates (mean±s.d.) are shown for each measurement.

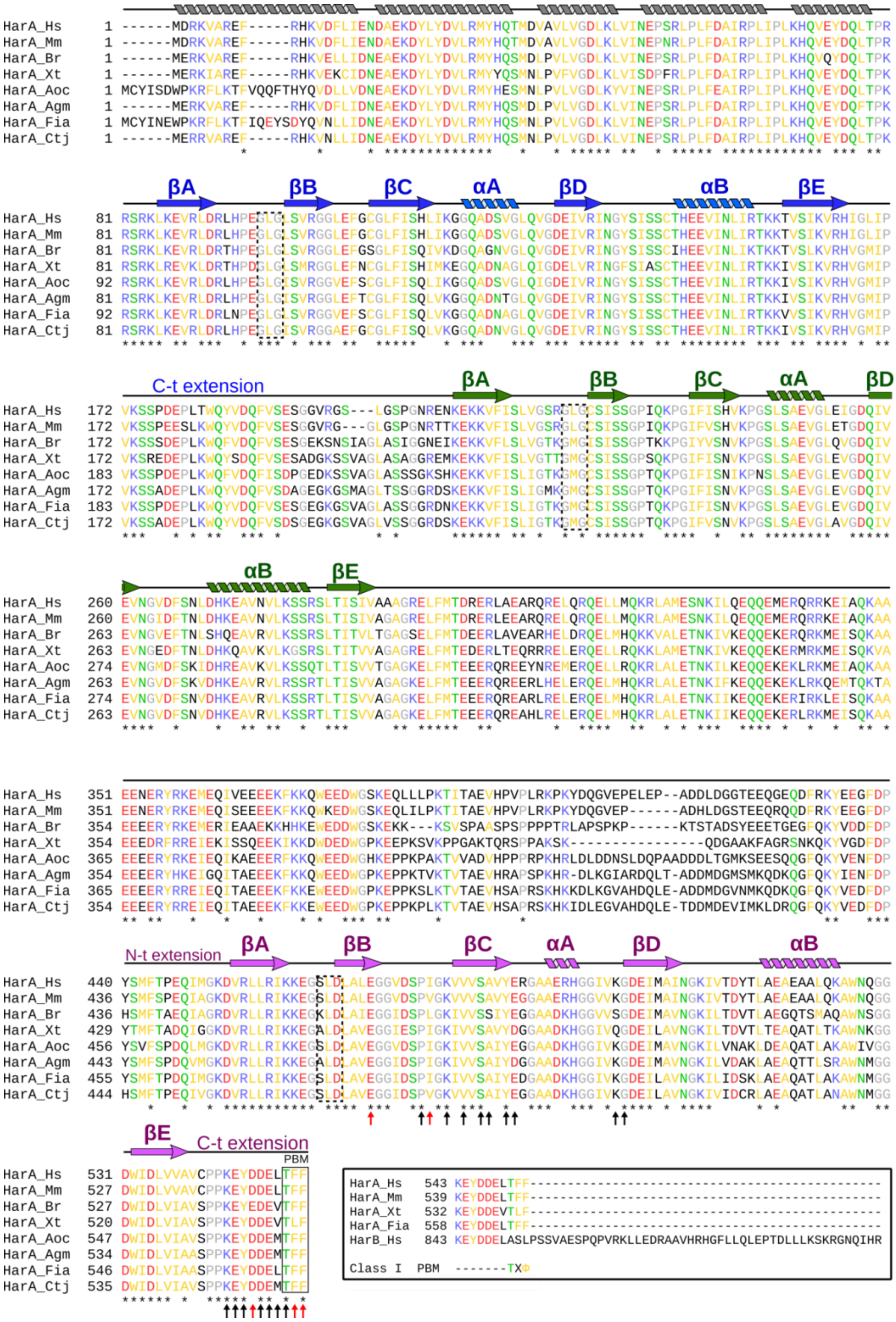

Supplementary Figure 5 – Harmonin-a is highly conserved in vertebrates

Alignment of harmonin-a from different vertebrates. Highly conserved residues are colored as in Supplementary Fig. 3. Residues strictly conserved are indicated with a star. The known secondary structural elements are shown above: grey (N-terminal domain), blue (PDZ1), green (PDZ2) and magenta (PDZ3). Dashed boxes highlight the canonical carboxylate-binding site of PDZ domains (the GLG motif in PDZ1 and PDZ2 binds class-I PBM while the SLD motif in PDZ3 could recognize class-II PBM[2]). The PBM in the C-terminal tail is also shown. Residues that participate in MF2 binding in the crystal structures are indicated with black arrows. Red arrows represent residues mutated and assayed for their effect on PDZ3c binding to Myo7a and Myo7b MF2. The box presents an alignment of the harmonin PDZ3 C-ter extension sequences compared to the human isoform b and a class I PBM. The Hs, Mm, Br, Xl, Aoc, Agm, Fia and Ctj abbreviations stand for *Homo sapiens* (RefSeq accession no. NP_001284693), *Mus musculus* (NP_001157205), *Brachydanio rerio* (NP_001035018), *Xenopus tropicalis* (NP_001096522), *Anolis carolinensis* (XP_016848700), *Alligator mississippiensis* (KYO22100), *Ficedula albicollis* (NC_021677) and *Coturnix japonica* (XP_01571962), respectively.

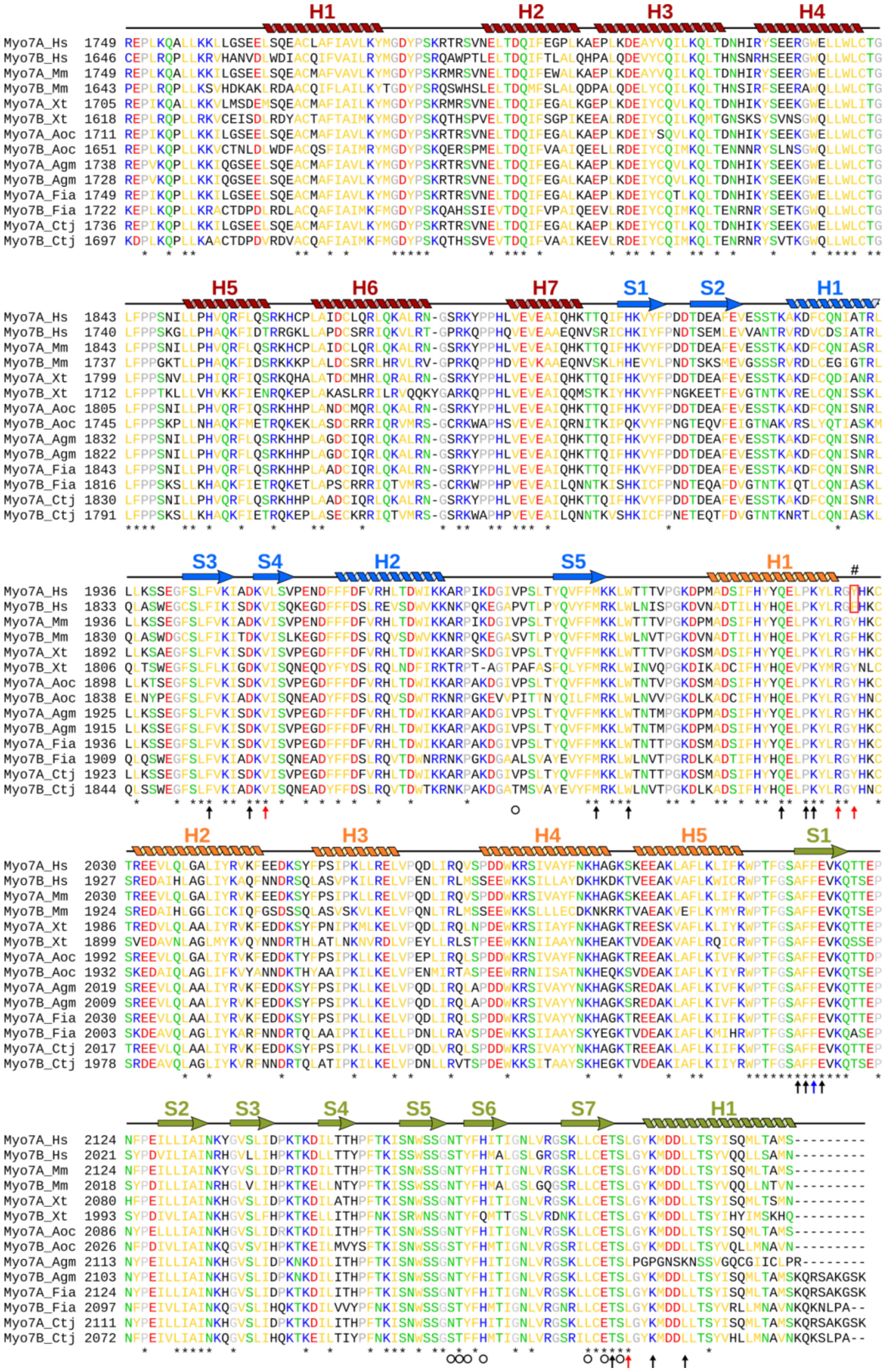

Supplementary Figure 6 – Structure based alignment of vertebrate Myosin 7a and b MF2

Conserved residues are colored as in Supplementary Fig. 3. The secondary structure elements of each lobe are indicated in red (MyTH4), blue (F1), orange (F2) and green (F3). Residues participating in the binding of the PDZ3 Cter are indicated with arrows, while residues interacting with the rest of the PDZ3 domain are indicated with circles. Red arrows correspond to residues found important for the binding of the PDZ3 Cter from mutation analysis. The Y2026/F1923 difference between Myo7a and Myo7b at the end of the F2 H1 helix that changes the positioning of the PDZ3 Cter and its interaction with the FERM domain is highlighted with a red box and a # symbol. As a result, the residue F2115/F2012 (blue arrow) interacts with the PDZ3 Cter in Myo7a but not in Myo7b. The Hs, Mm, Xt, Aoc, Agm, Fia and Ctj abbreviations stand for *Homo sapiens* (RefSeq accession numbers NP_000251 [Myo7a] and NP_001073996 [Myo7b], *Mus musculus* (NP_001243010, NP_115770), *Xenopus tropicalis* (XP_004912319, XP_002936659), *Anolis carolinensis* (XP_016852070, XP_016847423), *Alligator mississippiensis* (XP_019334939, XP_019341661), *Ficedula albicollis* (XP_016157295, XP_00505100) and *Coturnix japonica* (XP_01570798, XP_015726488), respectively.

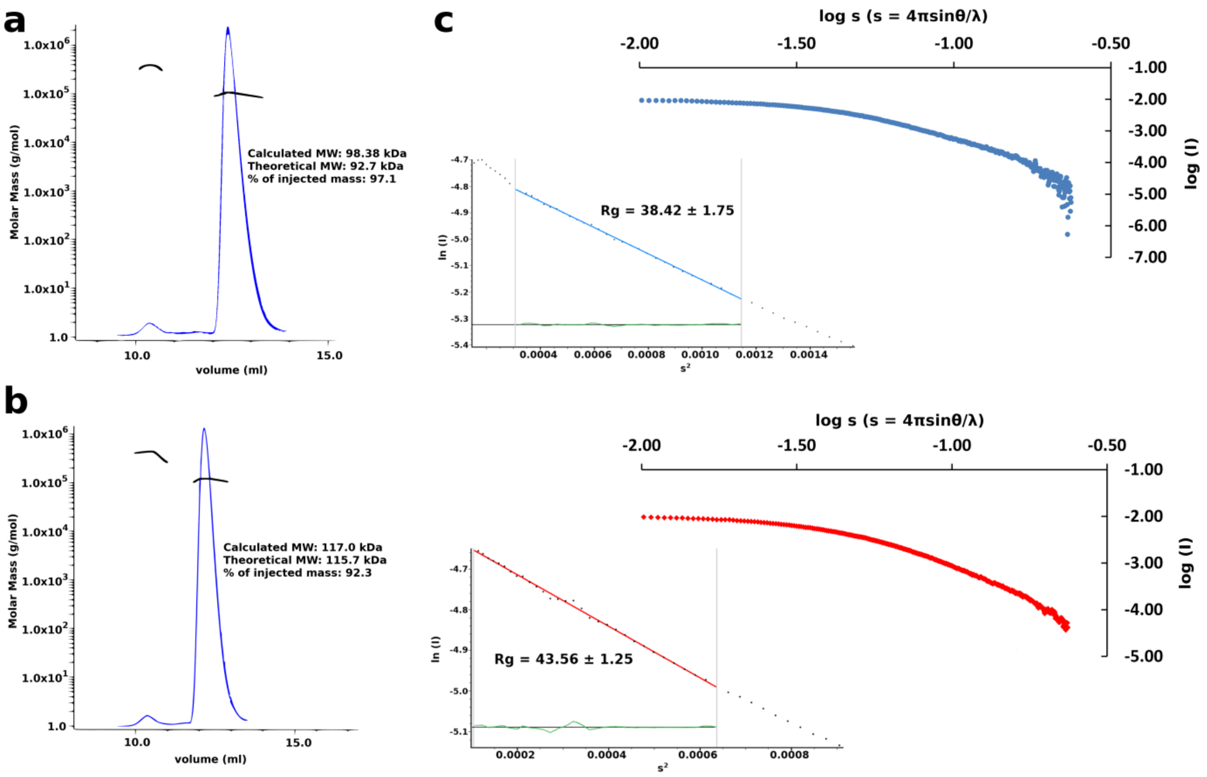

Supplementary Figure 7 – SEC-MALS and SAXS characterisation of members of the tripartite complex

(a) and **(b)**: SEC-MALS elution profiles displaying the plots of the refractometer (blue) and molar mass (black line) vs. elution time of the Myo7a MF1.SH3 + $SANS_{CEN-PBM}$ (a) and Myo7a MF1.SH3 + $SANS_{CEN-PBM}$ + NPDZ1 (b) complexes. **(c)** Full-Length (FL) Harmonin-a1 adopts a compact form in solution. Differences in the conformation of FL (top, blue) versus $FL_{\Delta Cter}$ harmonin (bottom, red) as determined by SAXS analysis.

Supplementary Table 1 – RMSD comparison between the Myo7b MF2 domain and other known MF structures.

PDB ID codes: Myo7a.MF1 (3PVL) – Myo7b.MF1 (5F3Y) – DdMyo7.MF1 (5EJY) – DdMyo7.MF2 (5EJR) – Myo10.MF (3PZD) – Myo7a.MF2 (this study, 5MV9) and Myo7b.MF2 (this study, 5MV8).

**MYTH4 domain (residues 1608-1797 in Myo7b) 145 Cα in the core + 45 Cα for the variable Nter extension**		
Myo7a MF2 (P1711-T1900)	0.466 for 124 Cα atoms	The MyTH4 core is well conserved. The major difference corresponds to the orientation of the last helix that interacts strongly and orients the F1 lobe (Myo10 and DdMF2). The Nter extension is different in Myo10, while strong similarities exist among all Myo7 MFs. Splice variants can drastically change the surface of this domain among Myo7 MF1 isoforms. This variability occurs on the MF surface in which the F3 lobe also participates.
Myo7b MF1	0.918 for 120 Cα atoms	
Myo7a MF1	0.795 for 100 Cα atoms	
Myo10 MF	1.892 for 100 Cα atoms	
DdMyo7 MF1	1.151 for 109 Cα atoms	
DdMyo7 MF2	1.355 for 101 Cα atoms	
**F1 domain (residues 1798-1892 in Myo7b, 93 Cα)**		
Myo7a MF2	0.579 for 73 Cα atoms	The fold of the F1 lobe core is well conserved. The major differences are seen in the H2 helix and the very flexible linker that precedes it. The linkers between the MyTH and F1, and the F1 and F2 lobes are quite variable allowing distinct relative position of these subdomains. The S1, S2, H1 helices are constrained by these inter-domain interactions while more dynamic variability is possible for the rest of the lobe (S3, S4, H2 helix and S5) allowing adaptability for the central groove of the FERM.
Myo7b MF1	0.953 for 65 Cα atoms	
Myo7a MF1	0.800 for 63 Cα atoms	
Myo10 MF	0.831 for **45** Cα atoms	
DdMyo7 MF1	0.898 for 61 Cα atoms	
DdMyo7 MF2	0.946 for **47** Cα atoms	
**F2 domain (residues 1904-2002 in Myo7b, 99 Cα)**		
Myo7a MF2	0.619 for 80 Cα atoms	The core of the F2 lobe is well conserved. It is formed by a bundle of helices connected by flexible loops. Note that Myo10 has a drastically different surface for this lobe due to longer H2 and H3 helices, a big insertion between them and a distinct conformation for the H3-H4 loop (18).
Myo7b MF1	2.888 for 75 Cα atoms	
Myo7a MF1	2.551 for 68 Cα atoms	
Myo10 MF	1.203 for 64 Cα atoms	
DdMyo7 MF1	7.389 for 83 Cα atoms	
DdMyo7 MF2	1.532 for 81 Cα atoms	
**F3 domain (residues 2010-2097 in Myo7b, 88 Cα)**		
Myo7a MF2	0.395 for 80 Cα atoms	The F3 fold is very well conserved. Some variability exists in the loops connecting the strands linked to both distinct sequence and flexibility. Note that DdMyo7 MF1 has a deformation in the S5 strand, preventing the ‘classic’ binding of FERM partners along this surface (18). In Myo7b MF1, the large rmsd compared to other F3 lobe is linked to a distinct orientation of the S5.S6.S7 beta sheet relative to the other structural elements.
Myo7b MF1	1.917 for **34** Cα atoms	
Myo7a MF1	1.635 for 58 Cα atoms	
Myo10 MF	0.979 for 59 Cα atoms	
DdMyo7 MF1	1.145 for 63 Cα atoms	
DdMyo7 MF2	1.301 for 74 Cα atoms	

Supplementary Table 2 – Analysis of 25 missense mutations in the Myo7a MF2 domain implicated in human deafness. The location of the mutations is shown in Fig. 4C.

Mutation	Domain	Predicted effect of the mutation	Reference
**R1861stop**		**Truncated myosin lacking MF2**	3
**E1716X**<br>**K1737fs**<br>**Q1798X**<br>**E1917X**<br>**G1942X**<br>**R2024X**			4
**Q2071X**			5
**C2182X**			6
**Y1719C**	MyTH4 N-ter linker<br>**Non pathogenic**	On the Nter extension of the MyTH4 domain.<br>Faces outside – or could be at the interface with the SH3 domain.	7,8,9
**R1743W**	MyTH4 N-ter linker	On the Nter extension of the MyTH4 domain.<br>Arginine faces the solvent – the tryptophan may disrupt the conformation of this Nter extension and potentially disrupt the interface with the SH3 domain of the Myo7 tail or with other unknown partners.	4,6
**A1770D**	MyTH4 H1 helix	internal – important for the folding of MyTH domain<br>Charge inside the hydrophobic core of the MyTH4 domain.	4
**L1799P**	MyTH4 H2 helix	internal – important for the folding of MyTH domain<br>Introduces proline in the middle of the helix – would destabilize the fold	10
**F1800I**	MyTH4 H2 helix	internal – important for the folding of MyTH domain<br>Substitution with a smaller residue inside the hydrophobic core of the MyTH4 domain. Low protein level in cells.	6
**E1812K/R**	MyTH4 H3 helix	internal – important for the folding of MyTH domain<br>Interaction with L1756. Disrupts interaction between N-ter linker and MF core.	11
**L1836P**	MyTH4 H4 helix	internal – important for the folding of MyTH domain<br>Pro in the middle of the H4 helix. Disruption of the folding.	12
**L1858P**	MyTH4 H5 helix	internal – important for the folding of MyTH domain<br>Pro inside the H5 helix. Disruption of the folding.<br>This is near the putative SANS binding site in the MyTH4.	4,13
**R1873W/Q**	MyTH4 H6 helix	Important for the MyTH/FERM F1 interface	14
**R1883Q**	MyTH4 H6-H7 linker	Important for the MyTH/FERM F1 interface<br>This is in the highly conserved RPE motif in the MyTH4-F1 interface.<br>R to Q will disrupt the interaction with E1891 and thus the correct folding of the MyTH4-F1 interface	4
**P1887L**	MyTH4 H6-H7 linker	Important for the MyTH/FERM F1 interface<br>This is in the highly conserved RPE motif in the MyTH4-F1 interface.<br>Introduction of a Leucine would disrupt the folding.	13
**V1918E**	F1 lobe – S2 strand	internal – important for the folding of F1 domain<br>Charge is introduced in the hydrophobic core of the F1 lobe.	15
**L1937P**	F1 lobe – end of helix 1	internal – important for the folding of F1 domain<br>Pro at this position would destabilize the folding of the F1 lobe.	16
**G1982R***	F1 lobe – disordered loop	**In a disordered variable loop – prevents harmonin PDZ3c binding.**	17
**G1982E**			18
**D2010N**<br>**Y2015H**	F2 lobe	internal – important for the folding of F2 domain<br>D2010 is involved in specific interactions that stabilize the F2 lobe.	4
**A2113P**	F3 lobe – S1 strand	internal – important for the folding of F3 domain<br>Pro at the beginning of the F3 strand would destabilize the folding.<br>**+ close to the binding site of the harmonin PDZ3-Cter**	19
**K2118N**	F3 lobe	**On the surface of the F3 lobe – close to the entrance of the groove. Prevents harmonin PDZ3c binding.**	20
**G2137E**	F3 lobe	internal – important for the folding of the F3 lobe<br>G2137 is in a small hydrophobic pocket. Glu will disrupt the folding.	21
**G2163S**	F3 lobe	**Glycine in the F3 strand that is the most used for binding partner interactions (integrin, DCC, selectin, CD43, ICAM-2…). Prevents harmonin PDZ3c binding.**	5,7,19, 22
**L2181P**	F3 lobe	internal – important for the folding of F3 domain<br>Introducing a proline at this position likely destabilizes the interactions between the beta strands and thus the integrity of the F3 lobe.	23
**T2184M**	F3 lobe	internal – important for the folding of F3 domain<br>Met would be directed in the hydrophobic core of the F3 lobe. There is no room for the side chain and this thus destabilizes the fold of the F3 lobe.	23
**L2186P**	F3 lobe	internal – important for the folding of F3 domain<br>Pro at the beginning of the F3 last helix. Not compatible with the folding.<br>**+ close to the binding site of the harmonin PDZ3-Cter**	24
**G2187D**	F3 lobe	internal – important for the folding of F3 domain<br>Asp is not compatible with the F3 lobe folding (clashes)	13
**A2204P**	F3 lobe	internal – important for the folding of F3 domain<br>This mutation would introduce a proline in the last F3 helix and would likely destabilize this helix and thus the F3 fold.	12

*** Note that due to differences in the organism and isoform, this mutation is mentioned as G1933R in the referred article**

Supplementary Table 3 – Primers and Synthesized Genes

Construct name	Residues
Myosin 7a (Q13402-2)*	
M7a MF1.SH3	F: taactcgaggatccggct<br>R: CTGCCTCTGATCGGGAGT
M7a MF2	F: GCGTGCCAAGCCCTACACGCTGGAGGAGTTTTCC<br>R: acctcgggctccgccgtGCCGCTGCTGTGATGATG
^{YFP}M7a MF2	F: acagctgaaCCCGAGGTGCGTGCCAAG<br>R: gccgctgctgtgatgatg<br>F: catcacagcagcggcgtgagcaagggcgaggagctg<br>R: gctgatcccggcggcggtcacgaa
M7a MF2 G1982R	F: CATCAAGGAC**cgt**ATTGTGCCCTC<br>R: GGCCGAGCTTTCTTTATC
M7a MF2 G1982E	F: TGTTCCCTCACTCACCTACCAG<br>R: AT**ttc**GTCCTTGATGGGCCGAGC
M7a MF2 K2118N	F: TCTTCGAGGTT**aac**CAAACTACGGAGCC<br>R: AGGCTGAGCCAAAGGTGG
M7a MF2 G2163S	F: CACGTACTTCCACATCACCATTGG<br>R: TT**gct**GCTGCTCCAGTTGGAGAT
Myosin 7b (Q6PIF6-1)	
M7b MF1.SH3	F: catatgGCGGAGGAGCCTGAGGAGG<br>R: ctcgagctaCCTCTTCTCTGGTGACATGG
^{YFP}M7b MF1.SH3	F: catcacagcagcggcgtgagcaagggcgaggagctg<br>R: GCCGCTGCTGTGATGATGATG
M7b MF1.SH3 A1128E	F: AAGCAGCCTG**gaa**CGGGGCTGGA<br>R: GTTTTGAAGTTCTCCGAGAGCTGC
M7b MF1.SH3 A1128E-R1129E-K1192E	F: AAGCAGCCTG**gaagag**GGCTGGATCC<br>R: GTTTTGAAGTTCTCCGAGAGC<br>F: GCAGGCTGTC**gaa**TCCAAGAAGCAC<br>R: AGCTCCAGCCAGGTGGG
M7b MF1.SH3 K1194E-K1195E-R1171E-R1172E	F: TGTCAAGTCC**gagg**AGCACATCCCC<br>R: GCCTGCAGCTCCAGCCAG<br>F: CGAGCGCCTG**gaagaa**ACCTATGCCAATGGGG<br>R: GCACAGAAGGGGCCGTAG
M7b MF2	F: AAGGAAAAGCTGCACACCC<br>R: ATGGCCGCTGCTGTGAT
^{YFP}M7b MF2	F: CATAAGGAAAAGCTGCACACCC<br>R: cagcttttccttatggctgccctgaaaatacaggttttc<br>F: catcatcacagcagcggcgtgagcaagggcgaggagc<br>R: gccgctgctgtgatgatgatg
M7b MF2 L2083W	F: CTGCTGTGCGAGACCTC**tgg**GGGCTATAAGATGGATGACC'
M7b MF2 R1921E-F1923W	F: CTGCCCAAGTACCTG**gaa**GGA**gtc**CACAAGTGTTCGCGGGAGGATGC
Harmonin-a1 (USH1C) (Uniprot: Q9Y6N9-1)	
Full length (FL)-harmonin	F: agatctATGGACCGAAAAGTGGCC<br>R: actagtctaGAAGAAGGTCAGCTCATCGTC
^{YFP}FL	F: agatctATGGACCGAAAAGTGGCC<br>R: actagtctaGAAGAAGGTCAGCTCATCGTC
FL ΔCter	F: **ggtgg**GCAGACGGCAACCACAAG<br>R: **agca**TAGACTAGTCGCGGCCGC
^{YFP}FL ΔCter	F: **ggtgg**GCAGACGGCAACCACAAG<br>R: **agca**TAGACTAGTCGCGGCCGC
NPDZ1	F: ggatccggagcagcaATGGACCGAAAAGTGGCC<br>R: ctcgagctaGCCTCGCACGCCCCCAGATT
^{YFP}NPDZ1	F: tagactagtttaactgctaacaaagcccgaaaggaag<br>R: GCCTCGCACGCCCCCAGA
NPDZ1.PDZ2	F: ggatccggagcagcaATGGACCGAAAAGTGGCC<br>R: ctcgagctaCCGGTCTGTCATGAACAG
^{YFP}NPDZ1.PDZ2	F: CCGGTCTGTCATGAACAG<br>R: tagactagtttaactgctaac
PDZ2-end	F: agatctGGCGTGCGAGGCAGCCTG<br>R: actagtctaGAAGAAGGTCAGCTCATCGTC
PDZ3c (isoA)	F: agatctCAGGATTTCCGGAAATA<br>R. actagtctaGAAGAAGGTCAGCTCATCGTC
^{YFP}PDZ3c (isoA)	F: agatctCAGGATTTCCGGAAATA<br>R. actagtctaGAAGAAGGTCAGCTCATCGTC
PDZ3c (isoA) F551V-F552V	F: CGTCTGCCCCCCAAAGGAGTATGACGATGAGCTGACC**gtcgtc**TA
PDZ3c (isoA) D546R-F551V-F552V	F: CGTCTGCCCCCCAAAGGAGTAT**agg**GATGAGCTGACC**gtcgtc**TA
^{YFP}PDZ3c (isoA) E469A	F: CTGGCCCTG**gca**GGCGGTGTG<br>R: GTCTAAGGATCCCTCCTTCTTGATGC
^{YFP}PDZ3c (isoA) I476W	F: GGACTCCCCC**tgg**GGGAAGGTGGTTGTTTCTG<br>R: ACACCGCCTTCCAGGGCC
^{YFP}PDZ3c (isoA) E469A-I476W	I476W mutation (above) introduced into ^{YFP}PDZ3c (isoA) E469A
PDZ3c$_{\Delta Cter}$	F: agcatagactagtcgcggccgc<br>R: **ggtgg**GCAGACGGCAACCACAAG
Harmonin-b3 (Uniprot: Q9Y6N9-5)	
PDZ3c$_{isoB}$	GGATCCTTAGACCTGGCCCTGGAAGGCGGTGTGGACTCCCCCATTGGGAAGGTG<br>GTTGTTTCTGCTGTGTATGAGCGGGGAGCTGCTGAGCGGCATGGTGGCATTGTGA

fragment synthesized by IDTDNA	AAGGGGACGAGATCATGGCAATCAACGGCAAGATTGTGACAGACTACACCCTGG<br>CTGAGGCTGACGCTGCCCTGCAGAAGGCCTGGAATCAGGGCGGGGACTGGATCG<br>ACCTTGTGGTTGCCGTCTGCCCCCCAAAGGAGTATGACGATGAGCTAGCTTCTCTT<br>CCCTCCTCCGTAGCTGAAAGCCCCCAACCGGTCCGAAAGCTCCTTGAAGACCGTGC<br>TGCCGTGCACAGACACGGGTTCCTCCTGCAGCTGGAGCCCACGGACCTTCTTCTGA<br>AGTCCAAAAGGGGAAACCAAATTCACCGTTAGACTAGT
SANS (USH1G) (Uniprot: Q495M9)	
SANS<br>full-length gene synthesized by GENSCRIPT	ATCTAGAATGGGACACCACCACCACCACCACATGAATGACCAATACCACCGTGCTG<br>CCCGCGACGGCTATCTGGAACTGCTGAAAGAGGCCACCCGTAAAGAACTGAACGC<br>TCCGGATGAGGACGGCATGACCCCTACTCTGTGGGCCGCTTACCACGGAAATCTG<br>GAGAGTCTGCGTCTGATCGTTTCGCGCGGTGGCGATCCCGACAAGTGCGATATTT<br>GGGGTAACACGCCACTGCATCTGGCGGCAAGCAATGGCCACCTGCATTGCCTGAG<br>TTTCCTGGTGTCGTTTGGCGCGAACATCTGGTGTCTGGATAATGACTACCACACTC<br>CCCTGGATATGGCCGCTATGAAAGGTCATATGGAGTGCGTCCGCTATCTGGACAG<br>CATCGCGGCAAAACAGAGCTCCCTGAACCCAAAGCTGGTTGGCAAGCTGAAAGAT<br>AAGGCCTTCCGTGAAGCTGAGCGTCGCATTCGCGAATGTGCCAAGCTGCAACGTC<br>GCCACCATGAACGTATGGAGCGTCGCTACCGTCGCGAACTGGCTGAGCGCAGCG<br>ACACCCTGTCCTTTTCTTCACTGACGAGTTCGACCCTGTCCCGTCGTCTGCAACACC<br>TGGCTCTGGGTTCTCATCTGCCTTATTCACAAGCGACTCTGCACGGTACGGCACGT<br>GGCAAAACCAAGATGCAGAAGAAACTGGAACGTCGCAAACAAGGAGGTGAGGG<br>TACTTTCAAAGTCAGCGAAGATGGCCGTAAGTCCGCACGCAGTCTGTCGGGACTG<br>CAACTGGGTTCCGACGTGATGTTCGTCCGTCAAGGAACCTACGCGAACCCCAAGG<br>AGTGGGGTCGTGCACCACTGCGCGATATGTTTCTGTCTGATGAAGACAGCGTTTCC<br>CGCGCAACTCTGGCCGCTGAACCTGCTCACAGTGAGGTGTCGACGGATAGCGGCC<br>ATGACTCACTGTTCACGCGTCCTGGTCTGGGCACTATGGTGTTTCGTCGCAACTAT<br>CTGTCTTCTGGACTGCACGGACTGGGTCGTGAAGATGGCGGACTGGACGGTGTG<br>GGAGCGCCGCGTGGTCGCCTGCAATCTTCACCTAGTCTGGACGATGACTCCCTGG<br>GTTCTGCAAATTCACTGCAAGACCGTAGCTGCGGCGAAGAGCTGCCGTGGGATGA<br>GCTGGACCTGGGTCTGGATGAAGACCTGGAACCCGAGACCTCTCCACTGGAGACT<br>TTCCTGGCCTCACTGCATATGGAAGATTTTGCGGCACTGCTGCGCCAGGAAAAAAT<br>TGACCTGGAGGCTCTGATGCTGTGTTCTGATCTGGACCTGCGTTCTATCTCAGTGC<br>CGCTGGGCCCTCGCAAGAAAATTCTGGGAGCCGTCCGCCGCCGCCGTCAAGCAAT<br>GGAACGCCCGCCCGCACTGGAAGACACTGAACTGTAACTGCAG
$SANS_{CEN\text{-}PBM}$	F: catatgGATAGCGGCCATGACTCACTG<br>R: ctcgagTTACAGTTCAGTGTCTTCCAG
$^{YFP}SANS_{CEN\text{-}PBM}$	F: catatgGATAGCGGCCATGACTCACTG<br>R: ctcgagTTACAGTTCAGTGTCTTCCAG
$ANKS4B_{CEN\text{-}PBM}$	F: catatgAGTGTGCACCATGAATCCATTC<br>R: ctcgagCTACAGGCTGGTGTCGACCAG
$^{YFP}ANKS4B_{CEN\text{-}PBM}$	F: catatgAGTGTGCACCATGAATCCATTC<br>R: ctcgagCTACAGGCTGGTGTCGACCAG

All primer sequences are listed from 5’ end to 3’ end, with nucleotides corresponding to sequences not encoded in the Myo7 or adapter gene (i.e. linkers or vector sequences) indicated in lowercase letters and altered codons in lowercase bold letters. Note that some codons were changed to facilitate cloning, in those cases the changes were non-mutagenic.

Supplementary Movie 1 – Myo7b MF2 FERM central cavity is very different from those of Myo7b and Myo7a MF1

Structural superimposition between Myo7b MF2 FERM (multi-colored), Myo7b MF1 (yellow) and Myo7a MF1 (violet) using the F1 lobe as a reference. The central cavity between the three FERM lobes (F1, F2 and F3) is drastically different between MF1 and MF2, explaining the binding selectivity between MF1 and MF2 partners.

Supplementary Movie 2 – Position of the 25 missense mutations found in Myo7a MF2

Representation of the missense mutations found in the MF2 of Myo7a (spheres). The spheres are colored following the color code used in Fig. 4C. The MF lobes are colored in red (MyTH4), blue (F1), orange (F2) and green (F3). The PDZ3c of Harmonin is in purple.

Supplementary References:

1. Yan, J., Pan, L., Chen, X., Wu, L. & Zhang, M. The structure of the harmonin/sans complex reveals an unexpected interaction mode of the two Usher syndrome proteins. *Proc. Natl. Acad. Sci. U. S. A.* **107,** 4040–5 (2010).
2. Siemens, J. *et al.* The Usher syndrome proteins cadherin 23 and harmonin form a complex by means of PDZ-domain interactions. *Proc. Natl. Acad. Sci. U. S. A.* **99,** 14946–14951 (2002).
3. Adato, A. *et al.* Mutation profile of all 49 exons of the human myosin VIIA gene, and haplotype analysis, in Usher 1B families from diverse origins. *Am.J.Hum.Genet* **61,** 813–821 (1997).
4. Jacobson, S. G. *et al.* Usher syndromes due to MYO7A, PCDH15, USH2A or GPR98 mutations share retinal disease mechanism. *Hum. Mol. Genet.* **17,** 2405–2415 (2008).
5. Brownstein, Z. *et al.* Novel myosin mutations for hereditary hearing loss revealed by targeted genomic capture and massively parallel sequencing. *Eur. J. Hum. Genet.* **22,** 768–775 (2013).
6. Liu, X. Z. *et al.* Mutations in the myosin VIIA gene cause a wide phenotypic spectrum, including atypical Usher syndrome. *Am. J. Hum. Genet.* **63,** 909–12 (1998).
7. Janecke, A. R. *et al.* Twelve novel myosin VIIA mutations in 34 patients with usher syndrome type I: Confirmation of genetic heterogeneity. *Hum. Mutat.* **13,** 133–140 (1999).
8. Cuevas, J. M. *et al.* Identification of three novel mutations in the MYO7A gene. *Hum. Mutat.* **14,** 181 (1999).
9. Boulouiz, R. *et al.* Analysis of MYO7A in a Moroccan family with Usher syndrome type 1B: novel loss-of-function mutation and non-pathogenicity of p.Y1719C. *Mol. Vis.* **13,** 1862–5 (2007).
10. Jiang, L. *et al.* Comprehensive molecular diagnosis of 67 Chinese Usher syndrome probands: high rate of ethnicity specific mutations in Chinese USH patients. *Orphanet J. Rare Dis.* **10,** 110 (2015).
11. Riahi, Z. *et al.* Whole exome sequencing identifies mutations in usher syndrome genes in profoundly deaf tunisian patients. *PLoS One* **10,** (2015).
12. Jaijo, T. *et al.* MYO7A mutation screening in Usher syndrome type I patients from diverse origins. *J. Med. Genet.* **44,** e71 (2007).
13. Bharadwaj, a K., Kasztejna, J. P., Huq, S., Berson, E. L. & Dryja, T. P. Evaluation of the myosin VIIA gene and visual function in patients with Usher syndrome type I. *Exp. Eye Res.* **71,** 173–181 (2000).
14. Roux, A.-F. *et al.* Survey of the frequency of USH1 gene mutations in a cohort of Usher patients shows the importance of cadherin 23 and protocadherin 15 genes and establishes a detection rate of above 90%. *J. Med. Genet.* **43,** 763–8 (2006).
15. Chen, Y. *et al.* Targeted next-generation sequencing in Uyghur families with non-syndromic sensorineural hearing loss. *PLoS One* **10,** e0127879 (2015).
16. Sodi, A. *et al.* MYO7A and USH2A gene sequence variants in Italian patients with Usher syndrome. *Mol. Vis.* **20,** 1717–31 (2014).
17. Riazuddin, S. *et al.* Mutation spectrum of MYO7A and evaluation of a novel nonsyndromic deafness DFNB2 allele with residual function. *Hum. Mutat.* **29,** 502–511 (2008).
18. Jacobson, S. G. *et al.* Retinal disease course in Usher syndrome 1B due to MYO7A mutations. *Investig. Ophthalmol. Vis. Sci.* **52,** 7924–7936 (2011).
19. Atik, T. *et al.* Comprehensive analysis of deafness genes in families with autosomal recessive nonsyndromic hearing loss. *PLoS One* **10,** (2015).
20. Naz, S. *et al.* Genetic causes of moderate to severe hearing loss point to modifiers. *Clin. Genet.* (2016). doi:10.1111/cge.12856
21. Lévy, G. *et al.* Myosin VIIA gene: heterogeneity of the mutations responsible for Usher syndrome type IB. *Hum. Mol. Genet.* **6,** 111–6 (1997).
22. Diaz-Horta, O. *et al.* Whole-exome sequencing efficiently detects rare mutations in autosomal recessive nonsyndromic hearing Loss. *PLoS One* **7,** (2012).
23. Yoshimura, H., Miyagawa, M., Kumakawa, K., Nishio, S. & Usami, S. Frequency of Usher syndrome type 1 in deaf children by massively parallel DNA sequencing. *J. Hum. Genet.* **61,** 1–4 (2016).
24. Bonnet, C. *et al.* Complete exon sequencing of all known Usher syndrome genes greatly improves molecular diagnosis. *Orphanet J. Rare Dis.* **6,** 21 (2011).
